# Shared vs separate structural representations: Evidence from cumulative cross-language structural priming

**DOI:** 10.1177/17470218231160942

**Published:** 2023-03-24

**Authors:** Danbi Ahn, Victor S Ferreira

**Affiliations:** 1 Neurobiology of Language Department, Max Planck Institute for Psycholinguistics, Nijmegen, The Netherlands; 2 University of California, San Diego, La Jolla, CA, USA

**Keywords:** Bilingualism, sentence production, cumulative structural priming, bilingual syntax

## Abstract

How do bilingual speakers represent the information that guides the assembly of words into sentences for their two languages? The shared-syntax account argues that bilinguals have a single, shared representation of the sentence structures that exist in both languages. Structural priming has been shown to be equal within and across languages, providing support for the shared-syntax account. However, equivalent levels of structural priming within and across languages could be observed even if structural representations are separate and connected, due to frequent switches between languages, which is a property of standard structural priming paradigms. Here, we investigated whether cumulative structural priming (i.e., structural priming across blocks rather than trial-by-trial), which does not involve frequent switches between languages, also shows equivalent levels of structural priming within- and cross-languages. Mixed results point towards a possibility that cumulative structural priming can be more persistent within- compared to cross-languages, suggesting a separate-and-connected account of bilingual structural representations. We discuss these results in terms of the current literature on bilingual structural representations and highlight the value of diversity in paradigms and less-studied languages.

## Introduction

Because any pair of languages will have differences as well as similarities, bilinguals need to represent different but highly related knowledge about the two languages they speak. Even so, proficient bilinguals are nearly flawless at using only their intended language and they do so nearly always correctly. This issue leads to a central topic in bilingualism: How is the knowledge of two languages organised in the one cognitive system of a bilingual speaker?

Studies have revealed that even when bilinguals speak in one language, linguistic representations (e.g., words and sounds) from both of their languages are accessed (for reviews, see Costa, 2005; Dijkstra & van Heuven, 2002; Kroll et al., 2017; Kroll & Gollan, 2014; Runnqvist et al., 2014). Recently, the field of bilingualism extended this topic to structural organisation in bilinguals, to investigate how comparable constructions in two languages are organised in a bilingual’s cognitive system. That is, both English and Spanish have comparable active (e.g., *the dog chases the cat; el perro persigue al gato)* and passive (e.g., *the cat is chased by the dog; el gato es perseguido por el perro*) constructions. For such comparable constructions, are structural representations in bilinguals’ two languages shared or separate?

One way that structural knowledge could be organised in bilinguals’ cognitive systems is that constructions could be completely separately represented. It may be helpful to have English and Spanish constructions fully separate, particularly when bilinguals are in a setting that requires them to use only one language (e.g., conversing with a monolingual friend). Given that there are still subtle differences in comparable constructions in English and Spanish (e.g., the word-to-word English translation of Spanish active *El perro persigue* ***al*** *gato* is approximately *the dog chases* ***at-him*** *the cat*, having a preposition before the patient), separate Spanish and English active constructions would be helpful in avoiding grammatical mistakes that resemble correct sentences of the non-target language, thereby aiding bilinguals when they want to speak in one language. On the contrary, having separate constructions for two languages is not the most economical way to represent sentence structures because some information is represented twice, leading to notable redundancy. Having separate structural representations for different languages might also present some inefficiency when switching between two languages often (e.g., during online language interpretating).

Alternatively, bilinguals might represent the analogous sentence structure across languages only once, which introduces the advantages and disadvantages that are opposite from those of having separate representations of sentence structures across languages. Specifically, although having the analogous construction represented only once reduces redundancy and might be useful when having to switch back and forth between languages often (e.g., translating from one language to another), it might be difficult to keep subtle differences straight which could lead to more grammatical errors when trying to speak in only one language.

This question of whether structural representations in the two languages bilinguals know are shared or separate has largely been investigated using cross-language structural priming methods. Structural priming (Bock, 1986; see Mahowald et al., 2016; Pickering & Ferreira, 2008) refers to the phenomena in which a current sentence is more likely to be repeated or more easily processed when it is structurally similar to a previous (“prime”) sentence. For example, in language production, English speakers are more likely to say *the ball is hit by the bat* after saying *the cat is chased by the dog* compared to after saying *the dog chases the cat*. The underlying idea is that after accessing one of the alternative constructions, speakers are more likely to use the same construction to describe another event rather than trying to access the other construction. The same idea applies to cross-language structural priming. If there is only one structure shared across languages, then speakers would be more likely to use the same construction that is already accessed in one language even when describing an event in another language. Crosslinguistic priming would not be observed if there are completely separate structural constructions for different languages, as eliciting one construction in one language would not necessarily elicit the similar construction in the other language.

Cross-language structural priming has been observed in multiple studies using different languages, providing evidence for shared structural organisation in bilinguals (e.g., Hartsuiker et al., 2004; Loebell & Bock, 2003; see Gries & Kootstra, 2017; Hartsuiker & Bernolet, 2017; Hartsuiker & Pickering, 2008; Kootstra & Muysken, 2017; Van Gompel & Arai, 2018). In these studies, bilinguals were given a priming sentence in one language and were asked to immediately produce a target sentence in another language. Bilinguals often used target sentence structures that matched the structure from the priming sentences, supporting an integrated (shared-lexicon, shared-syntax) account of bilingual language representation (Hartsuiker et al., 2004). This model claims that verbs in each language are connected to a *combinatorial node* (representing sentence structures that the verbs can take) that is shared between languages. Cross-language structural priming occurs because the combinatorial node remains more readily accessible after processing a sentence structure in one language, which then leads it to be reused during production of the analogous structure in another language.

**Figure 1.**
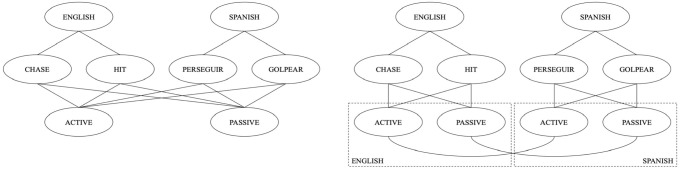
Shared (left) vs separate-and-connected (right) models of bilingual syntactic representation. *Perseguir* is Spanish translation for *to chase. Golpear* is Spanish translation for *to hit*. In both models, each lemma node (*chase, hit, perseguir*, or *golpear*) is connected to a relevant language node (English or Spanish) and both combinatorial nodes (active and passive). In the shared model, only one combinatorial node exists per sentence structure for both English and Spanish. In the separate-and-connected model, combinatorial nodes for the same sentence structure are represented twice, separately per language, but the combinatorial nodes that are analogous across languages are linked. *Source*. Adapted from Kantola and van Gompel (2011).

Although experiments using cross-language structural priming have been useful for better understanding bilingual structural representation, they do have several limitations. One is that models that assume separate and interacting syntactic representations between two languages could also explain the presence of structural priming across languages. That is, separate syntactic representations from different languages might be connected to each other when they represent the same or corresponding syntactic structures (much like translation equivalent lexical representations might be linked directly; Kroll & Stewart, 1994). Some studies have attempted to disentangle a shared-syntax model from a separate but closely connected model by comparing the strength of structural priming for within- vs cross-languages (Bernolet et al., 2013; Cai et al., 2011; Hartsuiker et al., 2016; Kantola & van Gompel, 2011; Schoonbaert et al., 2007). A shared-syntax account predicts no difference in the strength of structural priming across languages vs within a single language, because it claims that the representation of a given structure in a bilingual’s two languages is one and the same. In contrast, a separate but connected or interacting account of bilingual syntax predicts weaker structural priming across languages compared to within a single language, because activation must cross the extra connection between a combinatorial node for one language and a combinatorial node for a different language (see Figure 1).

Supporting a shared-syntax account, several studies found equivalent levels of structural priming within and across languages (Hartsuiker et al., 2016; Kantola & van Gompel, 2011; Schoonbaert et al., 2007). However, it is not completely conclusive that syntax must be shared between bilinguals’ two languages in part because such evidence is based on null effects (i.e., the lack of difference between the size of the two priming effects). For instance, it is possible that the separate syntactic representations from the two languages are so closely connected that the difference between within- and cross-language structural priming strength is hard to detect. Furthermore, in contrast to these studies, other studies reported stronger within- compared to cross-language priming (Bernolet et al., 2013; Cai et al., 2011; Travis et al., 2017). Bernolet et al. (2013) attributed these discrepancies to their participants’ low proficiency in the second language (L2), and argued that the development of shared syntactic representations with first language (L1) might require high proficiency in both languages.

Another possible reason why some studies have observed equivalent levels of structural priming within and across languages is that using standard prime–target structural priming to investigate the organisation of syntactic structures in bilinguals naturally involves using two languages at the same time. That is, in the same experimental session, participants need to switch between the two languages frequently (indeed, from prime to target). Although bilinguals can frequently switch between languages when speaking to another bilingual, doing so may require maintaining high accessibility of two languages even if they have separate representations. In other words, frequently switching between languages may increase the susceptibility of observing cross-language priming effects due to an increase in activation of the cross-language links between structures. Thus, to disentangle whether syntactic representations are shared or are separate and connected across two languages, examining bilingual speech during production of just one language will be valuable.

To do so, we adapted the cumulative structural priming method (Kaschak, 2007; Kaschak et al., 2006, 2011, 2014) which allows more separation between prime and target experimental trials and thus could provide an environment in which only one language is used without an expectation of frequent language switches. In a series of experiments with English monolingual speakers, Kaschak and colleagues found that patterns of experience with dative constructions affected the base rates of production of those constructions in a subsequent production block. For example, participants were asked to complete multiple sentence fragments that were designed to induce production of prepositional dative (PD) constructions (e.g., *Meghan gave the doll . . .*) in a block. Then, when asked to complete sentence fragments that could be completed as either PD or double object dative (DO) constructions (e.g., *The soldier gave . . .*) in a subsequent block, they were more likely to complete these sentences using PD constructions. In contrast, when participants were initially asked to complete sentence fragments that were designed to induce production of DO constructions (e.g., *Meghan gave her mother. . .*), they were more likely to complete the sentence fragments in the subsequent block using DO constructions. This suggests that structural priming might reflect the operation of the long-term implicit learning of the use of syntactic structures (e.g., Bock & Griffin, 2000; Chang et al., 2006). If syntactic structures are shared across languages, we should see a similar pattern of cumulative structural priming across languages as within. Alternatively, if syntactic representations from bilinguals’ two languages are separate (even if they are tightly connected), it is reasonable to assume that implicit learning in one language would not transfer to the other. If structural representations are separate across languages, we should observe less priming across languages than within.

Despite a rapidly growing number of standard cross-language structural priming studies, the evidence from cumulative structural priming is highly limited. Investigating the scope of implicit learning, Hwang and Shin (2019) tested Chinese–English bilinguals using cross-language cumulative structural priming. This study included two types of alternations with the same word order (dative) and different word orders (transitive) across Chinese and English. Interestingly, the cross-language cumulative priming effects from Chinese to English were observed only in transitive constructions, which have different word orders across Chinese and English. From this, the authors suggested a language-independent implicit learning mechanism unconstrained by surface word order. Furthermore, they attributed the lack of cross-language priming in dative constructions to different information structures, and argued that the representation of dative constructions might not be shared across Chinese and English. The mixed results from this study highlight the importance of using methods other than the standard structural priming method, especially with less-studied languages. More research involving various methods and typologically different languages is necessary to further understand structural representation in bilinguals.

Accordingly, we adapted the method from Kaschak’s studies and examined cumulative structural priming across languages in Korean–English bilinguals. Although Korean and English have different canonical word orders, the dative alternation has analogous features across the two languages. See (1) for examples of Korean dative sentences (NOM = nominative, DAT = dative, ACC = accusative, PRES = present tense, DECL = declarative; goal is boldfaced, theme is italicised).

(1) a. goal-theme, postpositional dative

아이가 **엄마에게** *선물을* 준다.

child-NOM **mother-DAT** *present-ACC* give-PRES-DECL

b. theme-goal, scrambled postpositional dative

아이가 *선물을* **엄마에게** 준다.

child-NOM *present-ACC* **mother-DAT** give-PRES-DECL

c. Acc-Acc, double-object dative

아이가 **엄마를** *선물을* 준다.

child-NOM **mother-ACC** *present-ACC* give-PRES-DECL

Both the sentences (1a) and (1b) have functional-level structures analogous to English PD construction (e.g., Baek & Lee, 2004; Urushibara, 1991; see also Shin & Christianson, 2009). Critically, however, the order of the theme (“present”) and the goal (“mother”) of the sentences can be interchanged in a way that (1a) has the same goal-theme order as English DO (e.g., The child is giving **her mother** *the present*), and (1b) has the same theme-goal order as English PD (e.g., The child is giving *the present* to **her mother**). In the Korean linguistics literature, sentence (1c) is argued to be analogous at the functional level to English DO structure (e.g., O’Grady, 1991). Supporting this claim, using a standard cross-language structural priming paradigm, some studies observed a structural priming effect from Korean to English between sentences (1a) and (1c) (Shin & Christianson, 2009; Son, 2020).

In the present study, we have selected the structures from (1a) and (1b) for the following reasons. First, the [Acc-Acc] pattern in (1c) is limited to a small subset of ditransitive verbs (*give, teach*, and *feed*; Jung & Miyagawa, 2004; O’Grady, 1991).^1^ Thus, sentences such as (1c) severely limit the type of experimental sentences and thus make it difficult to generalise the findings in structural priming beyond the few verbs. Considering the current rich literature on within-language structural priming using English, structural priming independent from specific verbs is necessary for stronger evidence of sharedness of a structure across languages. Furthermore, as discussed above, (1a) and (1b) are interestingly different in that they have the same functional-level structure but different surface-level word orders. The current evidence for the importance of surface-level word order for bilingual structural representation is mixed. While some studies found cross-language structural priming effects despite different surface-level word orders (e.g., Bernolet et al., 2009; Chen et al., 2013; Desmet & Declercq, 2006; Hartsuiker et al., 2016; Hwang et al., 2018; Hwang & Shin, 2019; Shin & Christianson, 2009), others did not find cross-language structural priming effects for constructions with different surface-level word orders (e.g., Bernolet et al., 2007; Jacob et al., 2017; Kidd et al., 2015; Loebell & Bock, 2003). If the surface-level word order alone can lead to implicit learning across languages, we should observe a cross-language cumulative structural priming between Korean and English using theme-goal vs goal-theme alternations.

If the representations of Korean and English dative constructions are completely shared across all processing stages, we should see that bilinguals are equally likely to use the constructions presented in a previous block regardless of whether the two blocks include the same or different languages. This result would provide a stronger support for previous studies which found equivalent levels of structural priming within and across languages (Hartsuiker et al., 2016; Kantola & van Gompel, 2011; Schoonbaert et al., 2007), showing a similar pattern of results even without an expectation of frequent language switches within an experimental session. If the constructions are completely separate, we should only observe within-language priming and not cross-language priming. If the constructions are separate but connected, we should observe structural priming both when the two blocks are the same language and when they are different languages, but less priming across languages. Any difference in the levels of structural priming within vs across languages should indicate differences in structural mechanisms across languages, suggesting that the structural representations of the two languages are not one and the same.

## Experiment 1

### Method

#### Participants

A total of 48 Korean–English bilinguals from the UC San Diego Department of Psychology subject pool volunteered for course credit or monetary compensation. The number of participants was decided based upon Kaschak et al. (2011) which found significant priming effects with 20 participants per condition. As we describe in the Materials and Design section, each of our participants was given four of the eight possible conditions, therefore allowing 24 participants per condition. All participants indicated that they were born and raised in Korea at least until the age of 11 years. All participants learned Korean as a first language and English as a second language, and all but six participants were dominant in Korean according to their ability to name pictures in each language (see below). All six participants who were dominant in English were highly proficient in both English and Korean (88.5% [1.5%] correct in English vs 83.6% [3.1%] correct in Korean on the picture-naming task, respectively). Detailed information about the participants’ language proficiency and language history is presented in Table 1.

**Table 1.** Participant characteristics and language proficiency based on self-report and modified MINT.

	Experiment 1 (*n* = 48)		Experiment 2 (*n* = 40)	
Current age	24.1 (4.4)		23.4 (3.8)	
Lived in the United States (years)	5.3 (3.2)		7.2 (3.8)	
	English	Korean	English	Korean
Age of acquisition (years)	8.8 (3.0)	0.1 (0.6)	8.0 (3.8)	0.2 (0.9)
Approximate percentage of daily use				
Current	49.9 (23.6)	49.0 (24.1)	54.7 (20.5)	44.0 (20.9)
Growing up	20.6 (16.5)	78.3 (17.6)	28.8 (18.9)	70.3 (19.5)
Proficiency self-rating (1–7)				
Listen	5.3 (1.2)	6.9 (0.3)	5.6 (1.2)	6.8 (0.6)
Read	5.3 (0.8)	6.8 (0.6)	5.5 (0.9)	6.7 (0.9)
Write	5.0 (0.9)	6.8 (0.7)	5.0 (1.1)	6.4 (1.2)
Speak	4.9 (1.2)	6.9 (0.4)	5.4 (1.2)	6.8 (0.6)
MINT (% correct)	78.1 (8.4)	86.8 (3.7)	79.5 (7.4)	84.9 (5.5)

All numbers represent means across participants. Standard deviations are indicated in parentheses. MINT: Multilingual Naming Test.

#### Materials and design

A total of seven ditransitive action verbs (e.g., *read*) were selected. For each action, 14 stock photos (98 photos total) depicting ditransitive actions with various agents, themes, and goals were selected from Shutterstock.com. To minimise bias towards theme-goal or goal-theme word order, whether the theme was on the left or right of the goal in the photos was counterbalanced.

Each picture was included only once. However, given the availability of pictures found on Shutterstock.com, many pictures depicted the same event (e.g., a woman reading a book to a girl). Sentences describing events in the pictures were created. Repetition of the same sentence was minimised using family relationships or synonyms that were reasonable given the photos. For example, photos depicting an event of a woman reading a book to a girl were described using different sentences such as, “The woman is reading the book to the girl,” “the mother is reading the bedtime story to her daughter,” or “the aunt is reading the fairy tale to her niece.” All items were translated into Korean.

Items were divided into 42 priming items and 56 target items. For each priming item, two sentence fragments that force the sentence completion to theme-goal (e.g., The woman is reading ______ to the girl.) or goal-theme (e.g., The woman is reading ______ the book) word orders were created. For each target item, one sentence fragment that could be completed using either theme-goal or goal-theme word orders was created (e.g., The man is giving ______).

Four lists were created. Each list included six blocks (two priming blocks and four target blocks). The priming items were presented in the first and the fourth blocks, and the target items were presented in second, third, fifth, and sixth blocks. Each priming block included 21 items, and each target block included 14 items. Each list involved one Korean priming block, one English priming block, two Korean target blocks, and two English target blocks. The priming sentence structures (theme-goal vs goal-theme) were different for the two priming blocks. The target blocks that were next to each other were always in different languages. Thus, each list involved both priming sentence structures (theme-goal vs goal-theme), both priming languages (Korean vs English), and both types of structural priming (cross-language vs within-language). The orders in which these were presented were counterbalanced across four lists (see the counterbalanced lists available at https://osf.io/6WRKM/).

**Figure 2.**
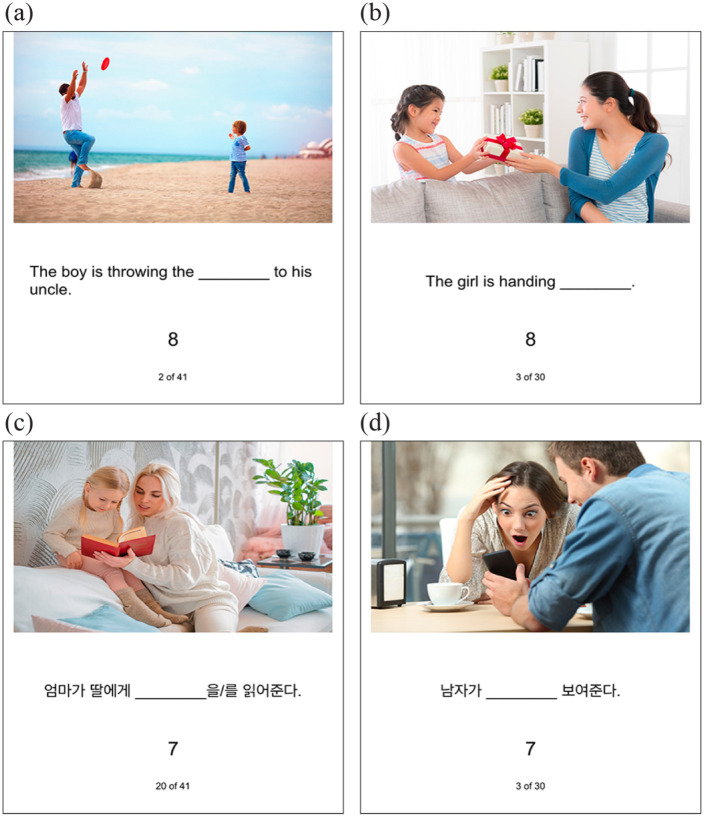
Example critical trials. Complete sentence materials for List 1 are available at tinyurl.com/2uj4zy29. (a) English theme-goal priming trial. (b) English target trial. (c) Korean goal-theme priming trial (mother-NOM daughter-DAT ________-ACC read-PRES-DECL). (d) Korean target trial (man-NOM ________ show- PRES-DECL). NOM: nominative; DAT: dative; ACC: accusative; PRES: present tense; DECL: declarative.

An additional 104 pictures depicting intransitive (e.g., sleep) or transitive (e.g., eat) events were selected for filler items. Sentences describing the events in the pictures were created using similar procedures as the critical items. However, the sentence fragments were created in a way that English sentence fragments involved blanks in the middle of the sentences when possible. This was to compensate for where the blanks appeared in the sentence fragments in the critical sentences. Because Korean is a verb-final language and all target sentences were given with the agents and verbs, all Korean critical target sentences had the blanks in the middle of the sentence fragments and all English critical target sentences had the blanks at the end of the sentence fragments. The filler items were inserted between critical items so that neither the critical items nor the filler items were presented more than twice in a row. Example materials are presented in Figure 2. All sentence materials are available at https://osf.io/6WRKM/.

#### Procedure

The experiment was presented on an iMac (21.5 inch, Mid 2014) using PsychoPy2 (Version 1.81.03; Peirce et al., 2019). Spoken responses were recorded via a Marantz PMD661 Solid State Recorder. Voice recordings were transcribed for later analyses.

Each trial was presented with a photo and a corresponding sentence fragment. Participants were asked to describe the photos (e.g., *a child drinking a green drink in a glass using a straw*) by filling in the blanks in the sentence fragments (e.g., *The child is drinking ______.*) using the language that the sentence fragments are presented in. During a pilot study, some participants had difficulty coming up with any words to complete sentences under time pressure, and claimed that they felt as though they had to come up with the “correct words” that fit in the blanks and could not think of them under time pressure. Such pilot participants were able to complete sentences in time when they were encouraged to start speaking as soon as they saw the pictures by saying anything. Thus, to support more sentence completions and to conceal the real purpose of the study, participants were assured that there was no one correct answer (e.g., *the drink* can be described as *the drink, the smoothie*, or *the juice*) and encouraged to use more than one word to fill in the blank using information present (e.g., *green, using a straw, from a glass*) or not present (e.g., *in the morning, delicious, healthy*) in the photo. To minimise possible goal drops, participants were encouraged to use all characters in their description of the photos when there was more than one character in the photo. Participants were told that the sentence fragments may be in English or Korean, but all trials in one block were in the same language.

Each experimental trial lasted for 8 s, with a countdown timer at the bottom of the screen. At the beginning of a trial, a photo and the sentence fragment appeared on the screen with a short click sound; the photo stayed on the screen for 8 s. At the end of the trial, a blank screen appeared for 200 ms. Trials in each block automatically advanced without a break. Participants were allowed a short break between each block. At the end of the experiment, participants completed an adapted version of Multilingual Naming Test (MINT; Gollan et al., 2012) and a language history questionnaire. To adapt the MINT for use in Korean, 7 items that are Korean–English cognates were excluded; thus, participants were tested on 61 items, first in English, and then in Korean. Note that the MINT was developed for use with speakers of Spanish, Chinese, Hebrew, and English, and the Korean adaptation was not validated against a Korean proficiency interview (as was done for the languages for which the MINT was originally developed). Thus, although it is not clear to what extent the scores accurately reflect degree of dominance in Korean vs in English, the scores are still useful for matching bilinguals within each language.

#### Coding and analysis

Only the responses from the target phase were used for analysis. Participants’ responses on critical items were coded into theme-goal, goal-theme, or other (all “other” responses were either goal drops or an absence of response). “Other” responses were removed prior to analysis (15% of the total data). Theme-goal responses were coded as 1 and goal-theme responses were coded as 0. Generalised linear mixed models (GLMMs) were fit using the “glmer” function from the lme4 package (Version 1.1-20; Bates et al., 2015) in R: A Language and Environment for Statistical Computing (Version 3.5.1; R Core Team, 2014). We used sum-to-zero contrasts (i.e., the intercept of the model was the grand mean of the dependent measure) to code the categorical predictors, language (English vs Korean), prime structure (goal-theme vs theme-goal), and prime type (across languages vs within languages). We first attempted to fit GLMMs incorporating the maximal random effects structure given the experimental design (Barr et al., 2013). For maximal models that did not converge, random effects accounting for the least variance were gradually removed until a model successfully converged. Using the R function “Anova” from the car package (Version 3.0-2; Fox & Weisberg, 2019), type III Wald chi-square tests were conducted to calculate main effects and interactions. When significant or marginally significant interactions were found, the emmeans package (Version 1.3.2; Lenth, 2019) was used to compute and compare estimated marginal means and standard errors for each treatment level. The data and R code, along with the converged random-effect structures and model output tables are available at https://osf.io/6WRKM/.

### Results

**Figure 3.**
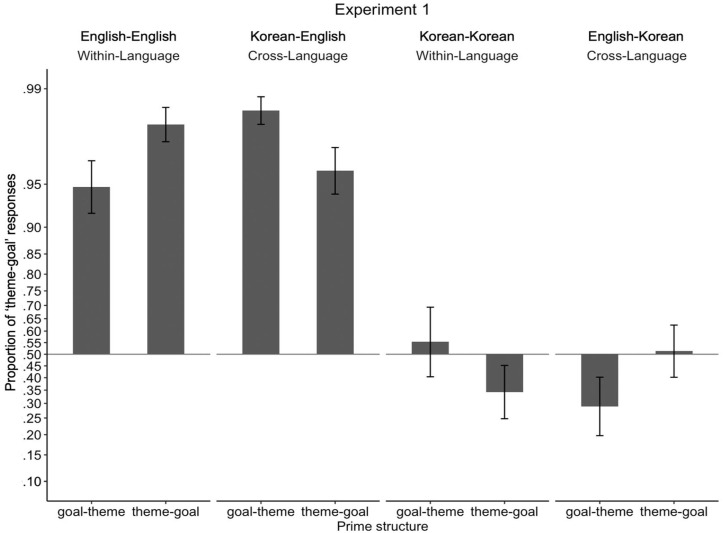
Proportion of “theme-goal” responses in Experiment 1. Proportions were transformed and presented in log-odds space to match statistical analyses, such that the vertical distances between proportions along the *y*-axis represent the magnitude of differences in log-odds space. Proportions for each participant were transformed to log-odds and then averaged. To allow the log-odds transformation, participants who had proportions of 0 or 1 were assigned a one-tenth of a proportion if they were to produce a single trial of another sentence structure (e.g., a participant who produced 0 theme-goal structure and 10 goal-theme structures was assigned a proportion of .01; a participant who produced 10 theme-goal structures and 0 goal-theme structure was assigned a proportion of .99). Error bars represent standard errors.

Figure 3 illustrates the proportion of theme-goal responses depending on target response language and conditions. Means are presented along with statistics, and standard deviations are reported in parentheses next to means. Although estimated marginal means were calculated following Chi-square tests which tested higher order interactions, we present estimated marginal means of the three-way interaction (language × prime structure × prime type) first for clarity of presentation.

Comparing estimated marginal means revealed that only English showed different within- vs cross-language priming effects. That is, when speaking English and the priming language was English, participants produced significantly more theme-goal responses when given theme-goal primes compared to when given goal-theme primes, showing a classic within-language structural priming effect in English (.95 [.14] vs .84 [.25], *b* = *−*2.23, *SE* = 1.13, *z* = *−*1.98, *p* = .048; transformed means as shown in Figure 1 = .98 vs .95). In contrast, when speaking English but the priming language was Korean, the rate of theme-goal responses was not influenced by theme-goal vs goal-theme primes, showing a lack of cross-language structural priming effect when the response language is English (.88 [.20] vs .97 [.06], *b* = 1.01, *SE* = .98, *z* = 1.03, *p* = .30; transformed means as shown in Figure 1 = .96 vs .99). Note that unexpectedly, not only did we fail to observe cross-language structural priming, but also the direction of priming was reversed such that the rate of theme-goal responses was numerically (but not statistically) higher when given goal-theme primes compared to given theme-goal primes. When speaking Korean and the priming language was Korean, the rate of theme-goal responses was not influenced by theme-goal vs goal-theme primes, showing lack of within-language structural priming when the response language is Korean (.43 [.32] vs .52 [.36], *b* = 0.63, *SE* = .77, *z* = .81, *p* = .42; transformed means as shown in Figure 1 = .34 vs .51). Similarly to the pattern of cross-language structural priming when English was the response language, note that the direction of priming was reversed. When speaking Korean and the priming language was English, the rate of theme-goal responses was not influenced by theme-goal vs goal-theme primes, showing lack of cross-language structural priming when the response language is Korean (.54 [32] vs .38 [.32], *b* = *−*1.05, *SE* = .66, *z* = *−*1.59, *p* = .11; transformed means as shown in Figure 1 = .51 vs .29).

Omnibus statistical analyses showed that participants produced significantly more theme-goal responses in English compared to in Korean (.91 [.17] vs .47 [.31], χ^2^(1) = 67.47, *p* < .001). Prime structure (theme-goal vs goal-theme) did not influence the response rates of theme-goal structure (.69 [.22] vs .69 [.23], χ^2^(1) = 1.71, *p* = .19). Prime type (within-language priming vs cross-language priming) did not influence the rates of theme-goal structure (.69% [.18%] vs .65% [.16%], χ^2^(1) < 1, *p* = .75). None of the two-way interactions, between prime structure and prime type, prime structure and language, and prime type and language, were significant (all χ^2^s <1). However, the three-way interaction between prime structure, prime type, and language was marginally significant, hinting that the extent of difference of within-language vs cross-language priming effects is different for English and Korean (χ^2^(1) = 3.71, *p* = .054).

### Discussion

Using a cumulative structural priming paradigm, we did not observe consistent cross-language or within-language structural priming. That is, we observed the expected structural priming effects in the English within-language condition, in which both prime and targets were in English. In all other conditions, we failed to find robust structural priming. Thus, taken at face value, these results are consistent only with fully separate structural representations between languages, though under such an account, it is unclear why we failed to observe Korean-to-Korean structural priming.

When Korean was the prime language, we observed numerical differences in the direction opposite to an expected priming effect, which is curious. This suggests that some aspect of our task setup might have worked against finding structural priming from Korean, for example, the way participants completed sentences. In particular, the priming and target items were created such that the blanks in the sentences were placed at about the same linear position across English and Korean. Although this was done to minimise the discrepancies in the English and Korean priming sentences, it may have had unintended effects. Recall that English theme-goal priming sentences involved participants filling in the theme of the sentence to complete the sentence (e.g., *The woman is reading ______ to the girl*). Because Korean is a verb-final language, having the blanks at approximately the same position across the two languages involved participants filling in the goal of the sentence to complete the Korean theme-goal priming sentences (e.g., *[woman] [book] _____ [is reading].*). This also happened in goal-theme priming sentences, such that participants had to come up with the goal of the sentence for English priming sentences (e.g., *The woman is reading _______ the book*), but had to come up with the theme of the sentence for Korean sentences (e.g., *[woman] [girl] _____ [is reading]*). Coming up with material for the blank might have driven the structural priming in this fill-in-the-blank paradigm rather than the entire sentence. For the Korean theme-goal priming conditions, having to come up with the goal of the sentence repeatedly might have put participants in “goal generating mode,” encouraging participants to come up with the goal of the sentence as soon as possible during the target phase, leading to more common goal-theme word order, accounting for the backward numerical differences. To test this hypothesis, we conducted a follow-up experiment in which materials were designed to elicit the theme for theme-goal priming conditions and the goal for goal-theme priming conditions for both the English and the Korean bias phases.

## Experiment 2

### Method

#### Participants

A total of 40 Korean–English bilinguals were recruited in the same way as in Experiment 1.^2^ All participants indicated that they were born and raised in Korea at least until the age of 11 years, except three participants who moved to the United States at the age of 4, 5, and 10 years, respectively. Most participants were dominant in Korean based on their scores on the modified MINT. A total of nine participants were highly proficient in both languages (85.1% [5.9%] correct in English vs 80.7% [7.1%] correct in Korean on the modified MINT, respectively). Detailed participant characteristics are provided in Table 1.

#### Materials and procedure

Materials and procedure were identical to Experiment 1, except the Korean and English sentence fragments from the bias phase encouraged sentence completions using the same thematic roles. For both the English and Korean theme-goal bias sentence fragments, blanks were presented where participants could complete the sentence by describing the theme of the sentence (e.g., *the woman is reading ______ to the girl; [woman] [girl] ________ [is reading]*). For both the English and Korean goal-theme bias sentence fragments, blanks were presented where participants could complete the sentence by describing the goal of the sentence (e.g., *the woman is reading ______ the book; [woman] ______ [book] [is reading]*).

#### Coding and analysis

The coding and analysis procedures were identical to Experiment 1. In total, 87% of the data were analysed.

### Results

**Figure 4.**
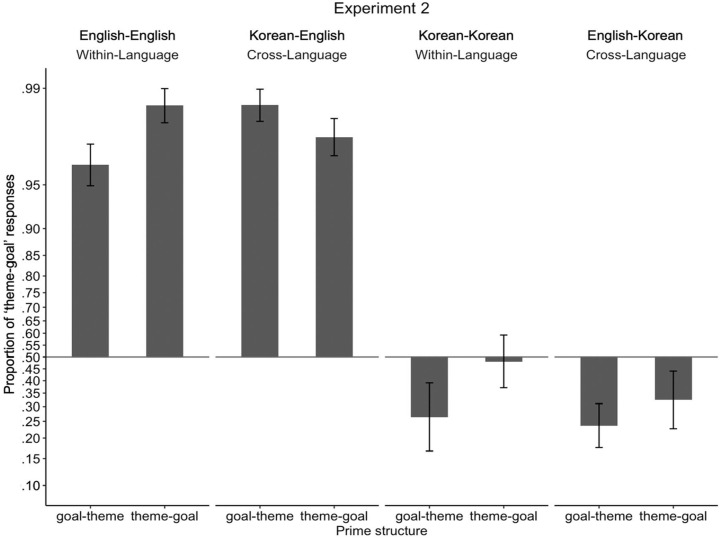
Proportion of “theme-goal” responses in Experiments 2. Proportions were transformed and presented in log-odds space to match statistical analyses, such that the vertical distances between proportions along the *y*-axis represent the magnitude of differences in log-odds space. Proportions for each participant were transformed to log-odds and then averaged. To allow the log-odds transformation, participants who had proportions of 0 or 1 were assigned a one-tenth of a proportion if they were to produce a single trial of another sentence structure (e.g., a participant who produced 0 theme-goal structure and 10 goal-theme structures was assigned a proportion of .01; a participant who produced 10 theme-goal structures and 0 goal-theme structure was assigned a proportion of .99). Error bars represent standard errors.

Figure 4 illustrates the proportion of theme-goal responses depending on target response language and conditions. Means are presented along with statistics, and standard deviations are reported in parentheses next to the means. Although estimated marginal means were calculated following Chi-square tests which tested higher order interactions, we present estimated marginal means of the three-way interaction (language × prime structure × prime type) first for clarity of presentation.

Comparing estimated marginal means revealed that only English showed different within- vs cross-language priming effects, and in different directions from Experiment 1. That is, when speaking English and the priming language was English, participants produced more theme-goal responses when given theme-goal primes compared to when given goal-theme primes, showing the expected within-language structural priming effect in English (.96 [.12] vs .92 [.10], *b* = *−*2.04, *SE* = .98, *z* = *−*2.09, *p* = .04; transformed means as shown in Figure 3 = .99 vs .96). In contrast, when speaking English but the priming language was Korean, the rate of theme-goal responses was not influenced by theme-goal vs goal-theme primes, showing a lack of cross-language structural priming effect when the response language is English (.95 [.08] vs .97 [.08], *b* = 0.63, *SE* = .93, *z* = .67, *p* = .50; transformed means as shown in Figure 3 = .98 vs .99). When speaking Korean and the priming language was Korean, the rate of theme-goal responses was not influenced by theme-goal vs goal-theme primes, showing lack of within-language structural priming when the response language is Korean (.51 [.33] vs .37 [.34], *b* = *−*0.79, *SE* = .69, *z* = *−*1.14, *p* = .26; transformed means as shown in, Figure 3 = .48 vs .26). When speaking Korean and the priming language was English, the rate of theme-goal responses was not influenced by theme-goal vs goal-theme primes, showing lack of cross-language structural priming when the response language is Korean (.37 [.30] vs .31 [.26], *b* = *−*0.34, *SE* = .50, *z* = *−*.68, *p* = .50; transformed means as shown in Figure 3 = .32 vs .24).

Omnibus statistical analysis showed that participants produced more theme-goal responses in English compared to in Korean (.95 [.09] vs .39 [.28], χ^2^(1) = 93.49, *p* < .001), and the same rate of theme-goal responses for within- vs cross-language priming (.69 [.18] vs .65 [.16], χ^2^(1) < 1, *p* = .68). In contrast to Experiment 1, participants produced more theme-goal responses when given theme-goal primes compared to when given goal-theme primes (.69 [.19] vs .65 [.17], χ^2^(1) = 5.45, *p* = .02), replicating classic cumulative structural priming. The structural priming effect was marginally affected by prime type (i.e., the interaction between prime structure and prime type was marginally significant; χ^2^(1) = 2.75, *p* = .10), such that collapsed across language, there were structural priming effects for within-language priming (.73 [.34] vs .64 [.37], *b* = *−*1.41, *SE* = .59, *z* = *−*2.40, *p* = .02), but not for cross-language priming (.66 [.36] vs .64 [.38], *b* = 0.14, *SE* = .49, *z* = .29, *p* = .77). The remaining two-way interactions between prime structure and language and prime type and language were not significant (both χ^2^ <1). In contrast to Experiment 1, the statistically marginal interaction between prime structure and prime type was not influenced by language (i.e., the three-way interaction between prime structure, prime type, and language was not significant; χ^2^(1) = 1.08, *p* = .30).

### Discussion

Similar to Experiment 1, Experiment 2 demonstrated the expected structural priming effects only in the English within-language condition, in which both prime and targets were in English. In all other conditions, we failed to find robust structural priming. In other words, only when the target language was English, the extent of structural priming was stronger for within-language compared to cross-language priming. In contrast, when the target language was Korean, not only was the extent of structural priming not different for within-language vs cross-language priming, but also we did not observe a standard within-language structural priming effect. From this, we might infer that although it is less clear how structural representations are accessed when Korean is the target language, structural representations in English and Korean are organised separately. Overall, it seems that structural priming from Korean, for both within-language and cross-languages, was weak and unreliable.

One reason why structural priming effects were inconsistent might be that the priming effects from the first priming phase (priming from Block 1 to Blocks 2 and 3) interfered with the priming efficacy of the second priming phase (priming from Block 4 to Blocks 5 and 6), especially given that cumulative within-language structural priming can persist as long as for a week between prime and target phases (Kaschak et al., 2011). That is, all participants received different types of priming structures in Block 1 vs Block 4 (e.g., all participants who received theme-goal priming structures in Block 1 received goal-theme priming structure in Block 4). In our analyses, we considered the effects in Blocks 5 and 6 to be structural priming only as a function of the priming structure in Block 4. If potential long-lasting priming effects from Block 1 interfered with the alternative structural priming in Block 4, priming effects in Blocks 5 and 6 would have been lost. If so, we might observe more consistent structural priming effects in Blocks 2 and 3 compared to when collapsing the results across all production blocks (as in the results presented above). To test this, we conducted a *post hoc* analysis.

#### Combined analysis of Blocks 2 and 3 from Experiments 1 and 2

The *post hoc* analysis was conducted only using the target phase trials following the first bias phase (Blocks 2 and 3). Data from Experiments 1 and 2 were combined to provide maximal power. GLMMs were fit following the same procedures as Experiments 1 and 2.

**Figure 5.**
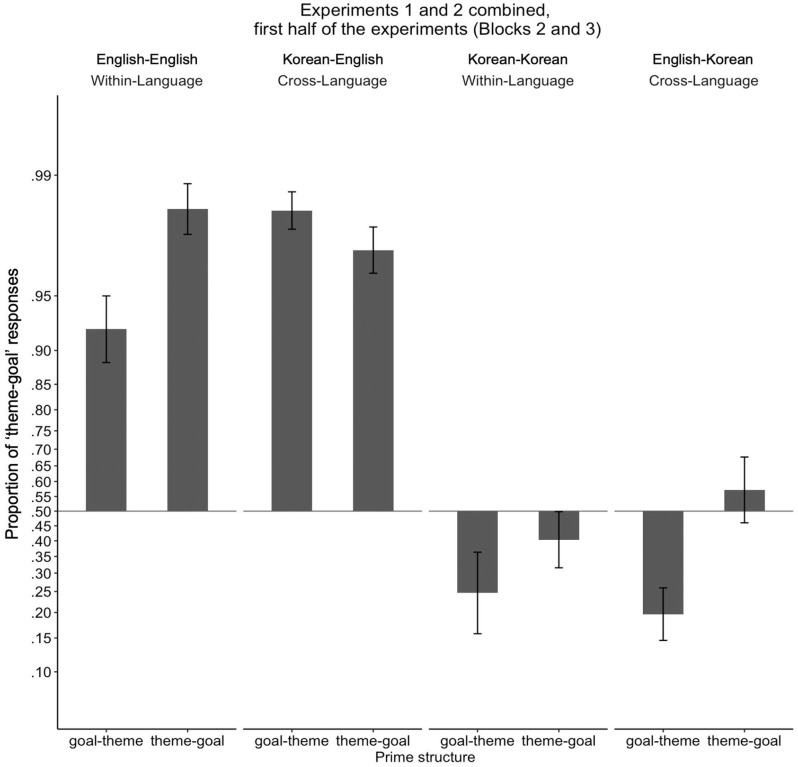
Proportion of “theme-goal” responses for Experiments 1 and 2 combined, for the first half of the experiments (Blocks 2 and 3). Proportions were transformed and presented in log-odds space to match statistical analyses, such that the vertical distances between proportions along the *y*-axis represent the magnitude of differences in log-odds space. Proportions for each participant were transformed to log-odds and then averaged. To allow the log-odds transformation, participants who had proportions of 0 or 1 were assigned a one-tenth of a proportion if they were to produce a single trial of another sentence structure (e.g., a participant who produced 0 theme-goal structure and 10 goal-theme structures was assigned a proportion of .01; a participant who produced 10 theme-goal structures and 0 goal-theme structure was assigned a proportion of .99). Error bars represent standard errors.

Figure 5 illustrates the proportion of theme-goal responses depending on prime structure and prime type, presented in log-odds space to match statistical analysis. Although estimated marginal means were calculated following Chi-square tests which tested higher order interactions, we present estimated marginal means of the three-way interaction (language × prime structure × prime type) first for clarity of presentation.

Comparing estimated marginal means revealed that English and Korean showed different patterns of within- vs cross-language priming effects. That is, when speaking English and the priming language was English, participants produced more theme-goal responses when given theme-goal primes compared to when given goal-theme primes, showing the expected within-language structural priming effect in English (.94 [.18] vs 82 [.24], *b* = *−*3.26, *SE* = 1.17, *z* = *−*2.78, *p* = .005; transformed means as shown in Figure 4 = .98 vs .92). In contrast, when speaking English but the priming language was Korean, the rate of theme-goal responses was not influenced by theme-goal vs goal-theme primes, showing a lack of cross-language structural priming effect when the response language is English (.94 [.10] vs .97 [.06], *b* = 0.84, *SE* = .63, *z* = 1.34, *p* = .18; transformed means as shown in Figure 4 = .97 vs .98). When speaking Korean and the priming language was Korean, the rate of theme-goal responses was not influenced by theme-goal vs goal-theme primes, showing lack of within-language structural priming when the response language is Korean (.45 [.30] vs .36 [.33], *b* = *−*0.59, *SE* = .65, *z* = *−*.91, *p* = .36; transformed means as shown in Figure 4 = .40 vs .25; because statistical significance was determined in log-odds space, and so the differences close to proportional extremes [0% and 100%] are statistically more prominent than differences around 50%; Jaeger, 2008). When speaking Korean and the priming language was English, the rate of theme-goal responses was significantly influenced by theme-goal vs goal-theme primes, showing the expected cross-language structural priming effect from English to Korean (.52 [.33] vs .27 [.25], *b* = *−*1.39, *SE* = .53, *z* = *−*2.62, *p* = .009; transformed means as shown in Figure 4 = .57 vs .20).

Omnibus statistical analyses showed that participants produced more theme-goal responses in English compared to in Korean (.92 [.17] vs .40 [.31], χ^2^(1) = 158.09, *p* < .001), and the same rate of theme-goal responses for within- vs cross-language priming (.64 [.36] vs .67 [.36], χ^2^(1) < 1, *p* = .49). Participants produced more theme-goal responses when given theme-goal primes compared to when given goal-theme primes (.70 [.20] vs .61 [.20], χ^2^(1) = 5.85, *p* = .02), replicating classic cumulative structural priming. The structural priming effect was significantly affected by prime type (i.e., the interaction between prime structure and prime type was significant; χ^2^(1) = 6.74, *p* = .009), such that collapsed across language, there were structural priming effects for within-language priming (.70 [.34] vs .59 [.37], *b* = *−*1.93, *SE* = .67, *z* = *−*2.87, *p* = .004; however, note from the above-mentioned estimated marginal means that this effect is largely driven by within-language structural priming in English), but not for cross-language priming (.73 [.32] vs .62 [.40], *b* = *−*0.28, *SE* = .41, *z* = *−*.68, *p* = .50). The remaining two-way interactions between prime structure and language and prime type and language were not significant (both χ^2^ <1). The interaction between prime structure and prime type was significantly influenced by language (i.e., the three-way interaction between prime structure, prime type, and language was significant; χ^2^(1) = 7.28, *p* = .007).

To summarise, the results from the *post hoc* analysis with only the first half of the experiments were similar to Experiment 2 results. However, we observed a few key differences. First, unlike in Experiment 2, where the interaction between prime structure and prime type was marginally significant, the *post hoc* analysis showed a significant interaction between prime structure and prime type, with significant within-language priming but non-significant cross-language priming. Moreover, unlike in Experiment 2, this *post hoc* analysis showed significant cross-language priming when the priming language was English and the target language was Korean. The two-way interaction of a subset of the data (with English-to-English vs English-to-Korean structural priming) was marginally significant (χ^2^(1) = 3.19, *p* = .07). Although we should be cautious about interpreting this marginally significant interaction, the English-to-English within-language priming trended towards a stronger structural priming effect compared to English-to-Korean cross-language priming. Thus, overall, the significant interaction in this *post hoc* analysis between prime type (within- vs cross-language) and prime structure suggests at the very least that English and Korean structural representations are separate; but the significant priming from English to Korean (along with the marginally stronger priming effect from English-to-Korean compared to from English-to-English) points to separate-but-connected representations of English and Korean structure, such that within-language priming will tend to be more robust than cross-language priming (but see General Discussion for more in-depth discussion about the unreliable priming effects with Korean).

## General discussion

Using a cumulative structural priming paradigm, two experiments examined the extent of within- vs cross-language structural priming effects when bilinguals do not switch between languages frequently. The same degree of structural priming for within- vs cross-language, even when there are no frequent language switches, would have provided strong support for the claims in the current literature that structural representations are shared between the two languages in bilinguals.

Experiment 1 showed that only when both the priming and target languages were English (i.e., only in English within-language structural priming), bilinguals produced more theme-goal sentences after theme-goal primes, showing the classic structural priming effect. Similar to Experiment 1, Experiment 2 showed that only when both the priming and target languages are in English (i.e., only for English within-language structural priming), bilinguals produced more theme-goal sentences after theme-goal primes, showing the expected structural priming effect. When the priming language was Korean and the target language was English, bilinguals did not show significant structural priming effects, showing the different extent of structural priming for cross-language compared to within-language effects. When the target language was Korean, we did not observe significant within- or cross-language priming effects. The *post hoc* combined analysis of Experiments 1 and 2 with only the priming blocks from the first half of the two experiments was consistent with the possibility that the priming effects from the first priming phase are long lasting and may have influenced the priming effects into the second half of the experiments. First, the two-way interaction between prime structure and prime type from this *post hoc* analysis was statistically significant, suggesting that the structural priming effect was stronger for within-language structural priming compared to cross-language structural priming. Second, an additional analysis of only the second half of the two experiments showed no priming effects in any conditions (see a detailed statistical report within the code at https://osf.io/6WRKM/). Given that the participants were given primes in different languages in the first half vs the second half of the experiment, this influence of primes from the first half of the experiment into the second half of the experiment further suggests the possibility of cross-language structural connectedness. That is, if the representations of the two languages are completely separate, a priming effect from one language in the first half of the experiment should not have had any influence on the priming effect from another language in the second half of the experiment. Altogether, on balance, these experiments and analyses show a robust within-language structural priming effect for English, and what is likely a weak between-language structural priming effect. This observation is difficult to reconcile with accounts that support a fully shared structural representations in bilinguals, but could support the claim of separate-and-connected structural representations in bilinguals (but see below for a more in-depth discussion about the unreliable priming effects with Korean).

The long-term nature of the priming assessed in this paradigm implies that any cumulative structural priming effect that is revealed relies on implicit learning. If the structural representations in the two languages are completely shared, this implicit learning in one language should be fully transferred to the other language. Given that we replicated Kaschak and colleagues’ findings of within-language structural priming in English, we might infer that English and Korean structural representations are separate rather than shared. Our results contrast with standard prime–target structural priming studies that showed the same extent of within- vs across-language structural priming and argued shared syntactic representations across languages in bilinguals (e.g., Hartsuiker et al., 2016; Kantola & van Gompel, 2011; Schoonbaert et al., 2007). Instead, our results are more consistent with the findings of stronger within- compared to cross-language structural priming, which support the separate and connected structural representations in bilinguals (e.g., Cai et al., 2011; Travis et al., 2017). In addition, our results suggest that shared structural representations do not automatically arise from high L2 proficiency (see Bernolet et al., 2013), given that our participants were highly proficient in English (their L2)—structural differences between languages may modulate proficiency effects.

One reason why we observed hints of evidence for separate structural representations might be the word-order differences between Korean and English. As we have discussed, although there are some similarities in Korean and English, there are structural differences between English and Korean as well. Most notably for the current sentences, the main verb is at different linear positions in English and Korean sentences, and articles are absent in Korean (e.g., for the PD, *Meghan gave the doll to the mother* vs *[Meghan][doll][mother][gave]*). Thus, although it may be possible for more typologically similar languages such as Dutch and English to have shared structural representations, languages with structural differences such as Korean and English might develop separate and connected structural representations instead. In fact, the current evidence for languages with different word orders is mixed. Using the same methods as the cross-language structural priming studies with languages with the same linear word order, several studies showed cross-language structural priming using structures with different linear word orders (e.g., Bernolet et al., 2013; Chen et al., 2013; Desmet & Declercq, 2006; Hwang et al., 2018; Muylle et al., 2020, 2021; Shin & Christianson, 2009; Weber & Indefrey, 2009), whereas other studies found cross-language structural priming only when the linear word order was the same across languages (Loebell & Bock, 2003; although they also did not observe a statistically significant within-language priming in German passive constructions). In an attempt to disentangle this mixed evidence, Ahn, Ferreira, and Gollan (2021) pointed at possible limitations of standard structural priming methods. That is, the cross-language structural priming effect in some studies for languages with different linear word orders might have been driven by the task properties of standard structural priming methods, rather than sharedness of structural representation. Bilinguals might simultaneously access both languages when expecting frequent language switches, either in anticipation of a language switch or as a result of a recent language switch. Cross-language priming effects could occur because both languages, along with the cross-language links, are active to support interleaved production of two languages, not because structural representations are shared across languages. Using a different method other than cross-language structural priming, Ahn et al. argued for separate representations of sentence structures in bilinguals. They tested Korean–English bilinguals while they produced noun phrases (“the cat above the piano”), which have different word orders in English and Korean (the Korean word order is [piano][above][cat]). Then, they examined the planning of each noun in a noun phrase by measuring articulation time of each word within an extended picture–word interference paradigm. They found that bilinguals plan English and Korean speech differently when describing events using noun phrases, suggesting that the representations of noun phrases are separate for English and Korean. In sum, there are discrepancies in the current literature on cross-language structural priming and evidence from a different paradigm that supports separateness of structural organisation for languages with different linear word orders. Importantly, our current study was designed to investigate bilingual structural representations with a paradigm that reduces the need to switch between languages often (unlike the standard structural priming methods). Thus, we might infer that our results provide additional insight to structural representation for languages with different linear word orders, such that Korean and English sentence structures are separately represented and that this pattern emerges more clearly when bilinguals do not expect frequent switches between languages.

Finally, it should be noted that the significant interaction between prime structure and prime type might have been driven at least partially by unreliable priming effects when Korean is the priming language. That is, in all analyses, including the *post hoc* analyses, we failed to observe a structural priming effect when Korean was the priming language. This absence of a priming effect persisted not only when the target language was English, but also when the target language was Korean. This was unexpected, especially given that other studies such as Shin and Christianson (2009) showed an equivalent degree of structural priming for English-to-English and Korean-to-English.

Shin and Christianson (2009) observed structural priming effects using theme-goal and Acc-Acc structures (analogous at the functional level to English goal-theme structures; e.g., O’Grady, 1991). Critically for the comparison to our study, the proportion of English theme-goal production after Korean theme-goal primes was numerically between the proportions of English theme-goal production after Korean goal-theme and Acc-Acc primes. In other words, Korean theme-goal sentences led to statistically non-significant priming in either direction of the English dative alternation. Thus, on the surface, it seems that our results replicate the findings from Shin and Christianson (2009) in that we also observed an absence of priming from goal-theme and theme-goal Korean primes. These results might suggest that Korean and English share structural representations at the functional level, and that the surface-level word order has minimal impact on this sharedness of the structural representation. However, another study using a standard structural priming method reported the cross-language structural priming that we initially predicted in our study: speakers used more English theme-goal sentences after Korean theme-goal primes compared to after Korean goal-theme primes (Son, 2020). With this and the English-to-Korean cross-language priming that we observed in the *post hoc* analyses, it is difficult to completely rule out the influence of surface-level word order on the structural representation of Korean–English bilinguals. It is possible that these conflicting results across different studies arise because of different mechanisms underlying standard and cumulative structural priming. While within-language priming of English shows similar results for both standard and cumulative structural priming, cross-language priming might tap into different mechanisms of standard vs cumulative structural priming, yielding seemingly inconsistent results across different types of structural priming studies. Thus, the cumulative structural priming paradigm may not be as effective in testing structural representations in Korean as it is in English. If such differences between standard and cumulative structural priming exist, the mechanistic differences might be more apparent especially with typologically different languages. Given that many inconsistencies in the literature come from studies with less-studied languages (e.g., Hwang & Shin, 2019; Shin & Christianson, 2009; Son, 2020), more studies with understudied languages would be valuable. In addition, it is worth noting that cumulative structural priming may be more prone to strategic effects, in a way that the blocked presentation of primes and targets can amplify even a slight preference for one sentence structure. This may explain the high proportion of theme-goal sentences observed in English. However, if such strategic effects exist, it is unclear why we only observed disproportionate preference of theme-goal sentences in English. One possibility is that L2 production is more sensitive to such strategic effects than L1 production. If so, we should observe that English native speakers who learned Korean as their L2 show high preference of one structure in Korean over the other in a cumulative structural priming paradigm.

Taken together, considering our mixed results, we might speculate that Korean dative sentences are represented differently from English dative sentences, and that this difference may not be observed in a standard structural priming method but is exaggerated in a cumulative structural priming paradigm. Furthermore, even though comparing estimated marginal means revealed that the within-language structural priming effect was statistically significant while the cross-language structural priming effect was not (and possibly stronger within- compared to cross-language structural priming when any English-to-Korean cross-language priming was observed), the higher order interaction between prime structure and prime type was only marginally significant. One reason might be that when speaking English, participants showed a strong bias for theme-goal compared to goal-theme structures such that it might have been difficult to observe robust priming effects, and even more difficult to observe differences in priming effects for within- vs cross-language conditions. Future research might use different sentence structures that show less bias for one construction over the other. Another possibility is that although we attempted to separate the production of each language by having separate prime and target blocks, the separation was not sufficient to counter possible dual-language activation similar to that in standard structural priming paradigms with two languages interleaved throughout an experimental session. Thus, even with some separation between the two languages, some lingering dual-language activation might have been enough to cause some cross-language structural priming. If so, we might expect that having the two languages further apart should dampen such cross-language structural priming if structural representations are separate. Given that cumulative within-language structural priming can last as long as a week between prime and target phases (Kaschak et al., 2011), a weakened structural priming effect for only cross-language priming should provide stronger support for separate structural representations of two languages.

In all, we presented possible data patterns that could point to separate-and-connected structural representations of Korean and English. This separateness may not be evident when using standard cross-language structural priming methods. Theoretical speculation over mixed results across the literature underlines that more studies using methods other than standard structural priming and understudied languages are crucial for a more complete understanding of bilingual structural representations.

## Notes

1 In the Korean target blocks across both experiments, 356 utterances (out of 2,464 Korean utterances) were coded as “other” responses (i.e., these responses were neither theme-goal nor goal-theme responses; see Coding and Analysis in Experiment 1 for a more detailed explanation). None of these “other” responses included [Acc-Acc] sentences, indicating that native Korean speakers do not spontaneously produce sentences such as (1c).

2 We planned to recruit 48 participants as we did in Experiment 1. However, the data collection was terminated early due to the COVID-19 lockdown. A total of 40 participants still allowed an even number of participants across the lists.
